# Prediction of adverse drug reactions based on pharmacogenomics combination features: a preliminary study

**DOI:** 10.3389/fphar.2025.1448106

**Published:** 2025-03-10

**Authors:** Mingxiu He, Yiyang Shi, Fangfang Han, Yongming Cai

**Affiliations:** ^1^ College of Medical Information and Engineering, Guangdong Pharmaceutical University, Guangzhou, China; ^2^ Department of Information, Guangdong Provincial Key Laboratory of Major Obstetric Diseases, Guangdong Provincial Clinical Research Center for Obstetrics and Gynecology, The Third Affiliated Hospital, Guangzhou Medical University, Guangzhou, China; ^3^ NMPA Key Laboratory for Technology Research and Evaluation of Pharmacovigilance, Guangzhou, China; ^4^ Guangdong Provincial Traditional Chinese Medicine Precision Medicine Big Data Engineering Technology Research Center, Guangzhou, China

**Keywords:** adverse drug reactions, comparative toxicogenomics database, chemical-gene interactions, gene-disease associations, convolutional neural networks

## Abstract

**Introduction:**

Adverse Drug Reactions (ADRs), a widespread phenomenon in clinical drug treatment, are often associated with a high risk of morbidity and even death. Drugs and changes in gene expression are the two important factors that affect whether and how adverse reactions occur. Notably, pharmacogenomics data have recently become more available and could be used to predict ADR occurrence. However, there is a challenge in effectively analyzing the massive data lacking guidance on mutual relationship for ADRs prediction.

**Methods:**

We constructed separate similarity features for drugs and ADRs using pharmacogenomics data from the Comparative Toxicogenomics Database [CTD, including Chemical-Gene Interactions (CGIs) and Gene-Disease Associations (GDAs)]. We proposed a novel deep learning architecture, DGANet, based on the constructed features for ADR prediction. The algorithm uses Convolutional Neural Networks (CNN) and cross-features to learn the latent drug-gene-ADR associations for ADRs prediction.

**Results and Discussion:**

The performance of DGANet was compared to three state-of-the-art algorithms with different genomic features. According to the results, GDANet outperformed the benchmark algorithms (AUROC = 92.76%, AUPRC = 92.49%), demonstrating a 3.36% AUROC and 4.05% accuracy improvement over the cutting-edge algorithms. We further proposed new genomic features that improved DGANet’s predictive capability. Moreover, case studies on top-ranked candidates confirmed DGANet’s ability to predict new ADRs.

## 1 Introduction

Adverse Drug Reactions (ADRs), commonly known as side effects (Research, 2022), have emerged as a major concern in public health and pharmacotherapy, imposing a substantial socio-economic burden along with severe incidence and mortality rates among patients during drug development. According to their mechanism of occurrence, ADRs can be divided into two main types: dose-related reactions (type A) and non-dose related or idiosynchrasic (type B), among which type A reactions are widely considered predictable (Micaglio et al., 2021). With the increasing availability of clinical and non-clinical data, computer algorithms have demonstrated the greatest utility in ADR prediction analysis. In the last 2 decades (2004∼2022), computer-based ADR predictions have primarily relied on the structural information of compounds (Das and Mazumder, 2023). However, the occurrence of Adverse Reactions (ARs) is not solely influenced by the structural information of the compound, but is also affected by the interaction between drugs or their intermediate metabolites and drug-effector gene-encoded proteins such as enzymes, receptors, ion channels, and the genes themselves. Studies have demonstrated that genetic variations in drug-metabolizing enzymes, drug transporters, and drug targets have a significant impact on changes in pharmacokinetics and pharmacodynamics of drugs (Zhou et al., 2015). Consequently, predicting ADRs solely based on the structural information of compounds may overlook critical information, potentially compromising the predictive performance of the model. According to recent investigations, pharmacogenomics accounts for ∼80% variability in drug pharmacokinetics and pharmacodynamics, as well as over 60% of ADRs (Cacabelos et al., 2019; Pirmohamed, 2023). For instance, HLA-pharmacogenomic markers are the main culprits that influence the mechanisms of immunopathogenesis of drug-induced severe cutaneous adverse drug reactions (SCARs) (Satapornpong et al., 2024). Currently the main clinical areas applying pharmacogenetic testing include hemolitc anaemias, malignant hyperthermia, porphyrias, severe skin disorders, Brugada and long QT syndromes (Micaglio et al., 2021; Satapornpong et al., 2024). Furthermore, changes in gene expression can often be detected prior to the emergence of histopathological changes or clinical signs (Zhang et al., 2020). This suggests that genes can serve as valuable predictive factors, providing early warnings and preventing the occurrence of ADRs, especially type B reactions (Micaglio et al., 2021). Consequently, integrating pharmacogenomic data with compound structural information into Machine Learning (ML) algorithms, rather than relying solely on compound structural information, could potentially enhance the timeliness and reliability of ADR predictions.

Several large-scale pharmacogenomics databases have recently become publicly available for research purposes, including the Library of Integrated Network-based Cellular Signatures (LINCS) L1000 project (Subramanian et al., 2017), Search Tool for Interactions of Chemicals (STITCH) (Kuhn et al., 2008), and the Comparative Toxicogenomics Database (CTD) (Davis et al., 2023). The LINCS L1000 project (Subramanian et al., 2017) profiled Gene Expression (GE) in cells treated with different dosages, with expressions assessed at various time points. The LINCS L1000 dataset has been widely used in recent studies to predict ADRs (Wang et al., 2016; Üner et al., 2023; Li et al., 2024) or Drug-Drug Interactions (DDIs) (Raja et al., 2017; Shankar et al., 2021). For instance, using the combination of the strongest GEs in LINCS L1000 and chemical structures of drugs, Wang et al. (2016) predicted ADRs using Extra Trees (ETs) (Geurts et al., 2006) classifiers (AUROC = 85.4%) and constructed an Adverse Drug Reaction-Gene Ontology (ADR-GO) network to link the most probable ARs predicted by the model to the relevant gene ontologies. Additionally, using the complete set of drug-perturbed Gene Expression Profiles (GEX) and their experimental Metadata (META), Üner et al. (2023) achieved a better predictive performance among five deep learning architectures, with Macro-AUC and Micro-AUC values of 79.0% and 87.7%, respectively. Given that the metadata is meaningless without GEs and could lead to numerous calculations, we chose the original GE feature for comparison in this study. STITCH is a resource for exploring known and predicted interactions of chemicals and proteins from 1,133 organisms. It integrates experimental, curated, and text-mined evidence, and users can filter their searches by tissue, affinity, and other criteria. Bongini et al. (2023) develop a model named DruGNN which constructed a graph to predict ADRs based on drug-protein interactions obtained from the STITCH database, and each protein was mapped to the gene from which it was derived. DruGNN achieved an accuracy of 86.3%. Recently, Li et al. (2024) proposed a novel model named BiMPADR, which integrated drug gene expression data extracted from the LINCS database into drug features and utilized gene–ADR associations extracted from the ADReCS-Target database (Huang et al., 2018) into ADR features to predict ADRs, achieving an AUC of 89.4%. While previous methods have demonstrated promising predictive outcomes, they exhibit limitations, including low AUROC scores, inability to apply to drugs with limited pre-existing information, and failure to consider both drug and ADR characteristics simultaneously. Moreover, these methods have not fully leveraged the potential of pharmacogenomics data, including the complex and diverse relationships between chemicals, genes, biomarkers, therapeutic targets, etc., rather than solely focusing on changes in gene expression. The CTD database houses a substantial amount of correlation data between chemicals, genes, phenotypes, and diseases. These data were meticulously organized and annotated by professional bioinformatics experts, ensuring data quality and accuracy. Despite the potential of pharmacogenomics data for ADR prediction, research in this area using the CTD database remains limited, possibly due to the challenges associated with data mapping. Consequently, there is still potential areas for advancement in ADR prediction utilizing pharmacogenomics data.

Although the above databases provide rich pharmacogenomics information, the enormous genetic data presents challenges to feature processing and model design for ADR prediction. Therefore, more efficient feature analysis is required to enhance the performance of prediction models. To achieve this goal, we creatively combined chemical structure descriptors, ADR semantic descriptors, and three different genomic descriptors [drug-perturbed GE changes, Chemical-Gene Interactions (CGIs), and Gene-Disease Associations (GDAs)] from various databases to establish drug-genomic-ADR relationships that can be used to train a Convolutional Neural Network (CNN)-based model for predicting drug-ADR associations.

Herein, we first constructed a benchmark validation dataset and five different features and then introduced a new deep learning method for ADR prediction. Subsequently, the ablation experiment validated the effectiveness of our proposed pharmacogenomics features, and additional case studies further demonstrated the practicality of our model as a predictor of novel ADRs.

## 2 Materials and methods

### 2.1 Benchmark datasets constructed using known drug-ADR relations

**FIGURE 1:**
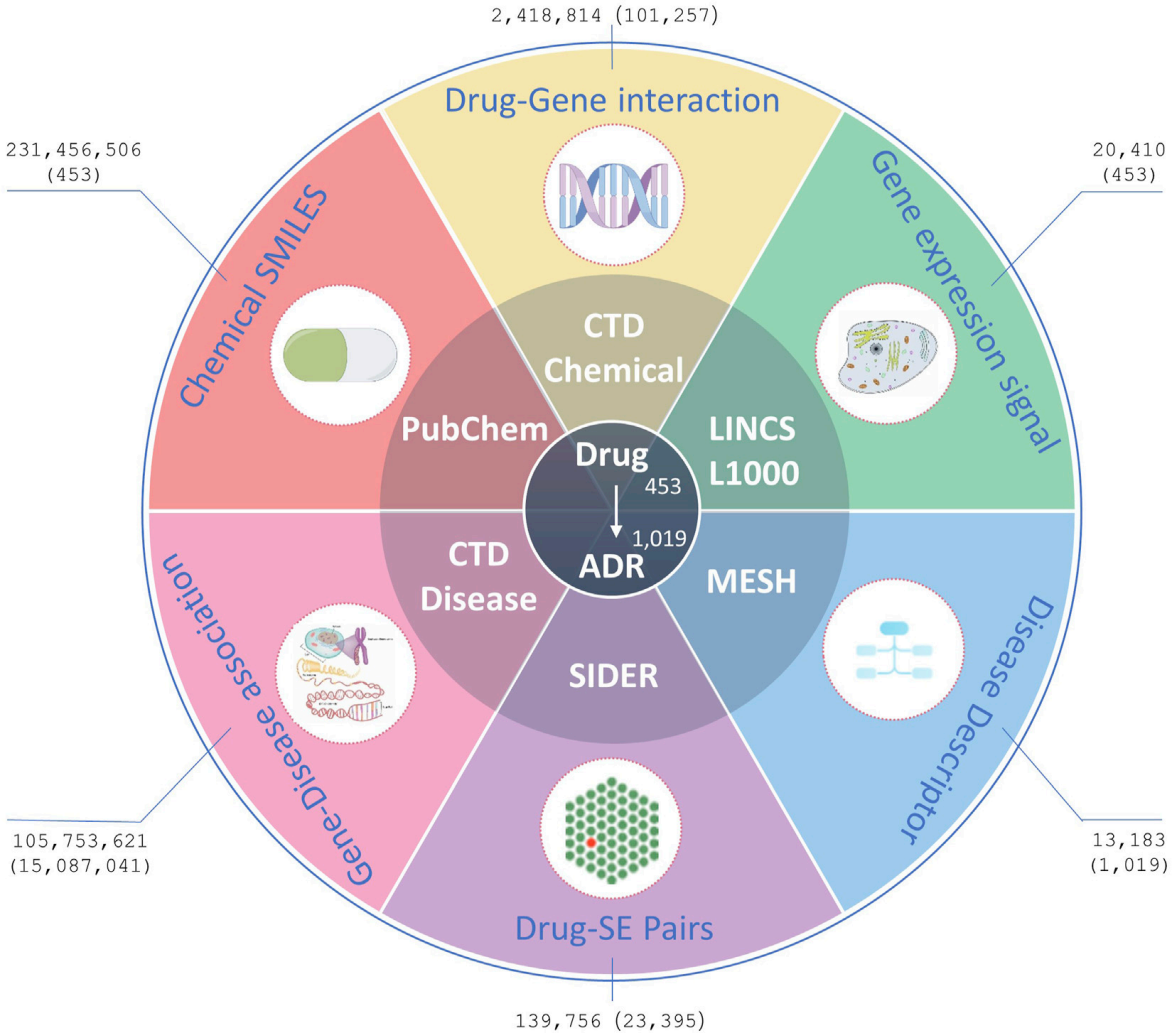
Data resources. Figures outside parentheses represent the number of original records downloaded from the databases, whereas figures inside parentheses represent the number of processed records finally used in the experiments.

The experimental benchmark datasets used in this study were from five public databases: Side Effect Resource 4.1 (SIDER) (Kuhn et al., 2016), LINCS L1000 (Subramanian et al., 2017), CTD (Davis et al., 2023), PubChem (Kim et al., 2019), and the US National Library of Medicine’s Medical Subject Headings (MeSH) (Fernandez-Llimos et al., 2017). In summary, SIDER was used to extract benchmark drug-ADR pairs, LINCS L1000, CTD and PubChem were employed to extract drug characteristics, MESH and CTD were utilized to extract ADR characteristics (Figure 1). In order to generate better results, drugs that are simultaneously recorded in CTD, PubChem, LINCS L1000, and SIDER databases and ADRs that are simultaneously recorded in CTD, MESH, and SIDER databases were included in this study. And all drugs or ADRs without pharmacogenomics data were also excluded. The details of data sets before and after processing in this study are shown in Table 1; Figure 1.

**TABLE 1:** Details of datasets before and after processing.

DataBase	Initial			Processed		
	Drugs	ADRs	Drug-ADR pairs	Drugs	ADRs	Drug-ADR pairs
CTD	175,287	13,183	—	453	1,019	—
LINC L1000	41,774	—	—	453	—	—
SIDER	1,430	5,868	139,756	453	1,019	23,395

The SIDER database is widely used for validating ADRs, and its current version contains 1,430 marketed drugs, 5,868 side effects, and 139,756 Drug-Side Effect Associations (DSAs). In SIDER database, drug terms are coded in STITCH compound IDs, which are also deformations of PubChem compound IDs. The compound IDs can be obtained by removing the prefixes. The Simplified Molecular-Input Line-Entry System (SMILES) strings and synonyms for all the drugs were bulk downloaded from PubChem using these compound IDs. Furthermore, the Chemical-Gene Interaction (CGI) and Gene-Disease Association (GDA) profiles were downloaded from CTD, including 175,287 drugs, 2,418,814 CGIs, 13,183 diseases, and 105,753,621 GDAs. The CGIs and GDAs were downloaded from CTD in March 2024. To reduce the dimensionality of data processing, CGI records were deduplicated using ChemicalID and GeneSymbol, irrespective of the Organism, Interaction, InteractionActions and PubMedIDs within the records. Similarly, the GDA records were deduplicated GeneSymbol and DiseaseID, irrespective of DirectEvidence, InferenceChemicalName, InferenceScore, OmimIDs, PubMedIDs within the records. Additionally, GE signatures for drugs/small molecule compounds in the landmark gene space were downloaded from maayanlab.net, originally processed from the LINCS L1000 database (Subramanian et al., 2017).

The five databases use different vocabularies to encode their drugs and ARs, which we mapped in various ways. The medications in CTD, PubChem, and LINCS L1000 which contained one or more SMILES were mapped using a 166-bit MACCS (Molecular ACCess System) fingerprint (Durant et al., 2002), which can be converted from any format of SMILES using the Python RDKit package (Landrum, 2024). The few drugs that could not be mapped with MACCS were mapped using names and synonyms retrieved from PubChem and CTD. The ADR terms in SIDER were mapped to Preferred Terms (PTs) coded in MedDRA v16.0. The MEDIC disease vocabulary of CTD is a modified subset of descriptors from the “Diseases” branch of MeSH combined with genetic disorders from the Online Mendelian Inheritance in Man (OMIM) database (Amberger and Hamosh, 2017). First, we downloaded MedDRA, SNOMED, and MeSH vocabularies from the Observational Medical Outcomes Partnership (OMOP) database (Sedlmayr et al., 2024). The MedDRA code was then mapped to the MeSH code using a standard concept ID applied in OMOP. Second, we downloaded the disease files from the Human Disease Ontology (HDO) database (Köhler et al., 2017), which contains the names, synonyms, and IDs linked to other data sources, including SNOMED, MeSH, and the Unified Medical Language System (UMLS) (Bodenreider, 2004), among others. The MedDRA terms in SIDER were matched to MeSH terms in CTD as long as they shared at least one linked ID. Not all ADRs can be mapped using linked IDs, hence, they were subsequently mapped using names and synonyms herein. Those that could not be mapped to MeSH were excluded from our study.

Among all the five attribute data sources, 453 drugs and 1,091 ADRs were finally used in our experiments, comprising 23,395 known drug-ADR pairs, 101,257 drug-gene interactions with 23,644 genes, and 15,087,041 GDAs with 53,968 genes. We constructed an algorithm for AR prediction using the multi-source data available on these management platforms.

### 2.2 Construction of drug similarity features

#### 2.2.1 Drug similarity based on chemical structure

The SMILES string representation for each drug structure was obtained from PubChem and then converted to topological fingerprints using the Python RDKit package (Landrum, 2024). Topological fingerprints are binary codes based on the topological configuration and rotational angles of quaternary rings within molecular structures. They are used to characterize molecule stereochemistry and potential interactions. Herein, the drug similarity matrices based on the Chemical Structure (CS) were expressed as: ${Sim}_{cs}^{drug-drug}$ ; and the Tanimoto Coefficient was used to measure the similarity score of each drug pair. The formula for calculating the similarity scores of drugs i and j was as follows:

$${Sim}_{cs\left(i,j\right)}^{drug-drug}=\frac{x_{i}^{cs}∙x_{j}^{cs}}{{\left\|x_{i}^{cs}\right\|}^{2}+{\left\|x_{j}^{cs}\right\|}^{2}-x_{i}^{cs}∙x_{j}^{cs}}$$

where $x_{i}^{cs}$ and ${\;x}_{j}^{cs}$ are the topological fingerprint representations of ${drug}_{i}$ and ${drug}_{j}$ , respectively.

#### 2.2.2 Drug similarity based on GE changes before and after drug perturbations

Herein, ${Sim}_{GE}^{drug-drug}$ was defined as the drug similarity matrix at the GE level. Based on works of Wang et al. (2016), we collected GE signature profiles perturbed by drugs/small molecule compounds from maayanlab.net. The Phase 1 experiment data of LINCS L1000 (GSE92742), in which a variable named “distil_ss” denotes the signature strength of every experiment, was the source data of the GE features. The larger the “distil_ss” variable, the more differentially expressed the landmark genes are within a signature. This approach quantifies the magnitude of the differential expression of landmark genes when comparing the average drug treatment to the DMSO treatment in the LINCS L1000 dataset. The “distil_ss” values were computed using the Characteristic Direction (CD) method (Clark et al., 2014), which generates gene expression signatures for drug perturbations in the 978 landmark gene space. The cosine similarity measure was used to compute the drug similarity based on gene expression changes. The formula was as follows:

$${Sim}_{GE\left(i,j\right)}^{drug-drug}=\frac{x_{i}^{GE}\cdot \;x_{j}^{GE}}{\left\|x_{i}^{GE}\right\|\left\|x_{j}^{GE}\right\|}$$

where $x_{i}^{GE}$ and $x_{j}^{GE}$ are the differential gene expressions of perturbations of ${drug}_{i\;}$ and ${drug}_{j}$ , respectively.

#### 2.2.3 Drug similarity based on GE changes before and after drug perturbations

Herein, CGIs were downloaded from CTD [ctdbase.org (last updated on 29th November 2023], which curates specific chemical-gene and chemical-protein interactions in vertebrates and invertebrates from published literature. Each CGI was quantified based on four degrees: Increases (e.g., “Chemical X increases the expression of Gene Y mRNA”), decreases, affects, or does not affect. Interactions with the “does not affect” degree were excluded from CTD. There were also numerous indirect CGIs [e.g., “Chemical X inhibits the reaction (protein P results in the increased expression of Gene Y)”]. The variable “InteractionAction” which has 3,431 distinct values, was used to categorize the interactions.

Data dimensionality was first lowered by encoding drug-gene interactions as one-hot vectors without considering cell types and interaction degrees to reduce computational complexity. Briefly, for each drug, all genes that interact or do not interact with it were labeled as 1 and 0, respectively. Subsequently, gene sets ${GT}_{i}=\left\{g_{i1},g_{i2},g_{i3},\ldots ,g_{in}\right\}$ which contain n genes that interact with drug $d_{i}$ and gene sets ${GT}_{j}=\left\{g_{j1},g_{j2},g_{j3},\ldots ,g_{jm}\right\}$ which contain m genes that interact with drug $d_{j}$ were obtained. The more identical the genes that interact with the two drugs ( $d_{i}$ and $d_{j}$ ), the higher their similarity. In other words, the similarity between the two drugs, $d_{i}$ and $d_{j},$ can be quantitatively determined using the intersection and union ratio of the two gene sets, ${GT}_{i}$ and ${GT}_{j}$ . The Jaccard index was used to calculate the drug similarity ${Sim}_{CGI}^{drug-drug}$ based on CGIs, and the formula was as follows.

$${Sim}_{CGI\left(i,j\right)}^{drug-drug}=\frac{\left|{GT}_{i}\cap \left.{GT}_{j}\right|\right.}{\left|{GT}_{i}\cup \left.{GT}_{j}\right|\right.}$$

### 2.3 Construction of ADR similarity features

#### 2.3.1 ADR semantic similarity extracted from the MeSH database

**FIGURE 2:**
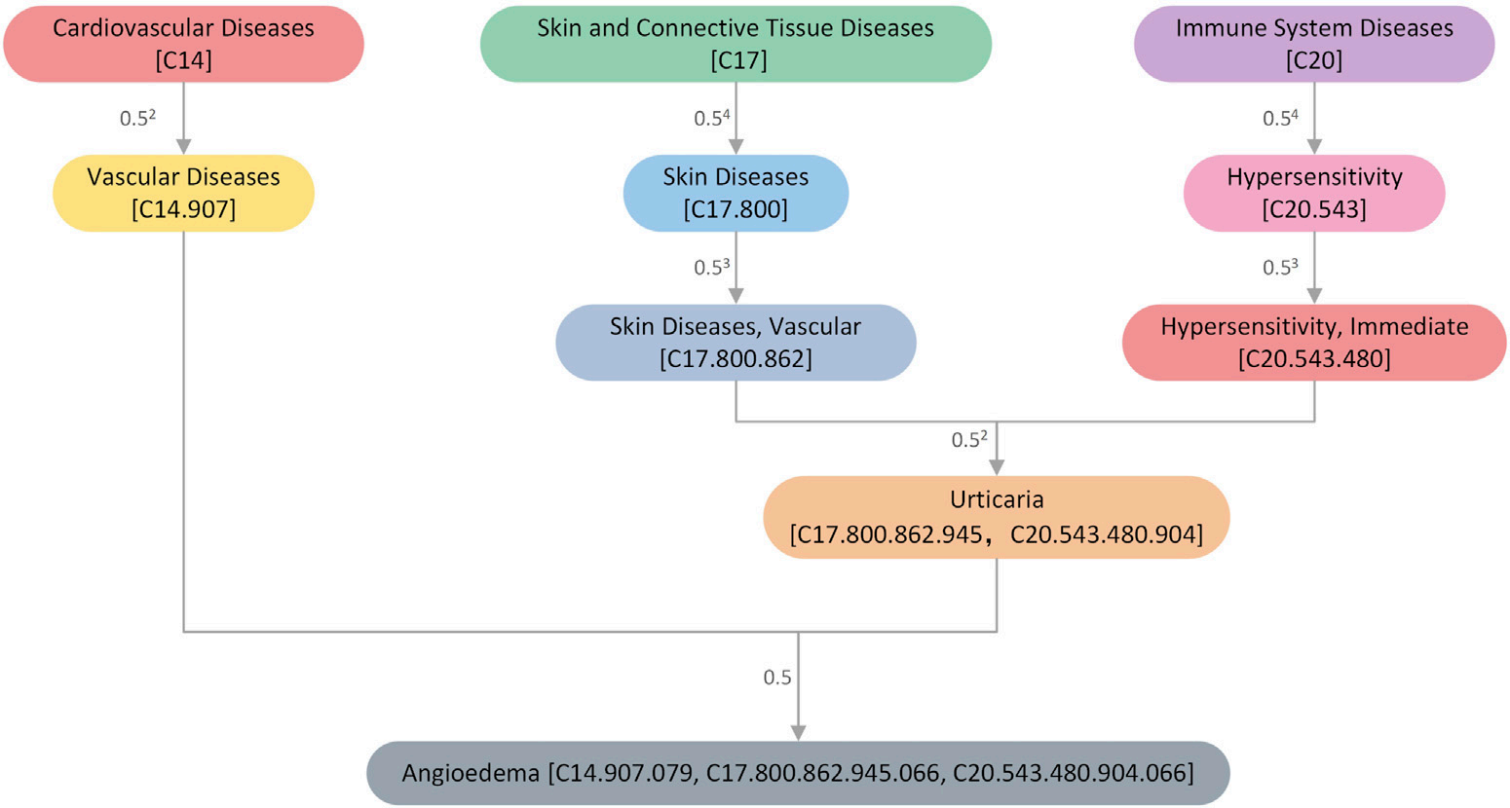
An illustration of the hierarchical structure of the disease “Angioedema” extracted from the MeSH database. The numbers next to the connecting line indicate the size of the semantic contribution factor. The closer it is, the more significant the contribution factor.

Drug-induced diseases are a subset of ADRs (Pathan et al., 2018) and are also commonly referred to as side effects. Herein, we attempted to map all ADRs to a hierarchical clinical terminology vocabulary to describe them more professionally. All the ADRs in the SIDER database were mapped to the MeSH database, as earlier mentioned in Section 2.1. The MeSH database is a commonly used standard medical thesaurus published by the National Library of Medicine (NLM) in the United States. It comprises hierarchical sets of descriptors based on their semantic categories and subject attributes. The MeSH database allows for the searching of diseases at various levels of specificity and can be used to examine disease correlations. For instance, the “Angioedema” entry has three possible addresses or codes: C14.907.079, C17.800.862.945.066, and C20.543.480.904.066, which belong to Cardiovascular Disease [C14], Skin and Connective Tissue Disease [C17], and Immune System Disease [C20] categories, respectively. Figure 2 shows the hierarchical structure of ' Angioedema’ extracted from the MeSH database.

We constructed a Directed Acyclic Graph (DAG) for each ADR using hierarchical descriptors from MeSH, with nodes representing ADR descriptors and edges representing the relationship between the current ADR and its ancestor ADRs. Each ADR, s, can be presented as a graph, ${DAG}_{s}=\left(s,N_{s},E_{s}\right)$ , where $N_{s}$ is the set of all ancestor nodes, including the ADR node ( s ) itself, and Ed is the set of parent nodes pointing to the child node edges. The semantic contribution value of a node (n) to an ADR (s) in ${DAG}_{s}$ can be calculated as follows:

$$C_{s}\left(n\right)=\left\{\begin{array}{c} 1,if\;n=s\\ \max\left\{\Delta *C_{s}\left.\left(n^{\prime }\right)\right|n^{\prime }\in children\;of\;n\;\right\},otherwise \end{array}\right.$$

where Δ is a semantic contribution factor for the edges linking a node ( n ) to its child ( $n^{\prime }$ ). Herein, Δ was set to 0.5. By summarizing all nodes that an ADR (s) has, we can obtain the semantic value of ADR s, $\text{DV}\left(s\right),$ as follows:

$$DV\left(s\right)=\sum_{n\in N_{s}}C_{s}\left(n\right)$$

Where $N_{s}$ is the set of the ADR node ( s ) and its ancestor nodes. Notably, ADRs with more identical ancestors often have greater similarity. Herein, we defined ${Sim}_{{MESH}_{\left(i,j\right)}}^{side-side}$ as the semantic similarity between ADRs, $s_{i}$ and $s_{j}$ , and it was calculated as follows:

$${Sim}_{{MESH}_{\left(i,j\right)}}^{side-side}=\frac{\sum_{n\in N_{s_{i}}\cap N_{s_{j}}}\left(C_{s_{i}}\left(n\right)+C_{s_{j}}\left(n\right)\right)}{DV\left(s_{i}\right)+DV\left(s_{j}\right)}$$

#### 2.3.2 ADR similarity based on GDA

In this study, GDAs were obtained from CTD. There are three types of direct evidence for a GDA: M marker, Mechanism, or T therapeutic. As in ${Sim}_{CGI}^{drug-drug}$ , we used gene sets ${GS}_{i}=\left\{g_{i1},g_{i2},g_{i3},\ldots ,g_{in}\right\}$ to denote n genes with associations with ADR $s_{i}$ , and gene sets ${GS}_{j}=\left\{g_{j1},g_{j2},g_{j3},\ldots ,g_{jm}\right\}$ to denote m genes with associations with ADR $s_{j}$ . Diseases associated with more identical genes tend have greater similarity. In this regard, GDA-based ADR similarity, ${Sim}_{{GDA}_{\left(i,j\right)}}^{side-side}$ , can be measured using the Jaccard index as follows.

$${Sim}_{{GDA}_{\left(i,j\right)}}^{side-side}=\frac{\left|{GS}_{i}\cap \left.{GS}_{j}\right|\right.}{\left|{GS}_{i}\cup \left.{GS}_{j}\right|\right.}$$

### 2.4 Drug-gene-ADRs network for ADR prediction

After data preprocessing in Sections 2.2, 2.3, we obtained three ( ${Sim}_{cs}^{drug-drug}$ , ${Sim}_{GE}^{drug-drug}$ , and ${Sim}_{CGI}^{drug-drug}$ ) and two ( ${Sim}_{MESH}^{side-side}$ and ${Sim}_{GDA}^{side-side}$ ) similarity indices for drugs and ADRs, respectively. We then constructed the input vector of drug as Drug and Side as ADR as follows:

$$Drug=\left[{Sim}_{CS}^{drug-drug},{Sim}_{GE}^{drug-drug},{Sim}_{CGI}^{drug-drug}\right]$$

$$Side=\left[{Sim}_{MESH}^{side-side},{Sim}_{GDA}^{side-side}\right]$$

We constructed feature crosses using the Cartesian product to better understand the nonlinear relationship between drug features and ADRs. Feature crosses are a useful feature engineering technology that can help the model capture nonlinear relationships in the data. According to research, feature crosses can better capture the interaction between features than fully connected operations (Lian et al., 2018). Herein, the feature crosses of Drug and Side were defined as $V_{FCs}$ and illustrated as follows:

$$V_{FCs}=Drug\;⨯\;Side=\left\{\left(d,s\right)\left|d\in Drug,s\in Side\right.\right\}$$

**FIGURE 3:**
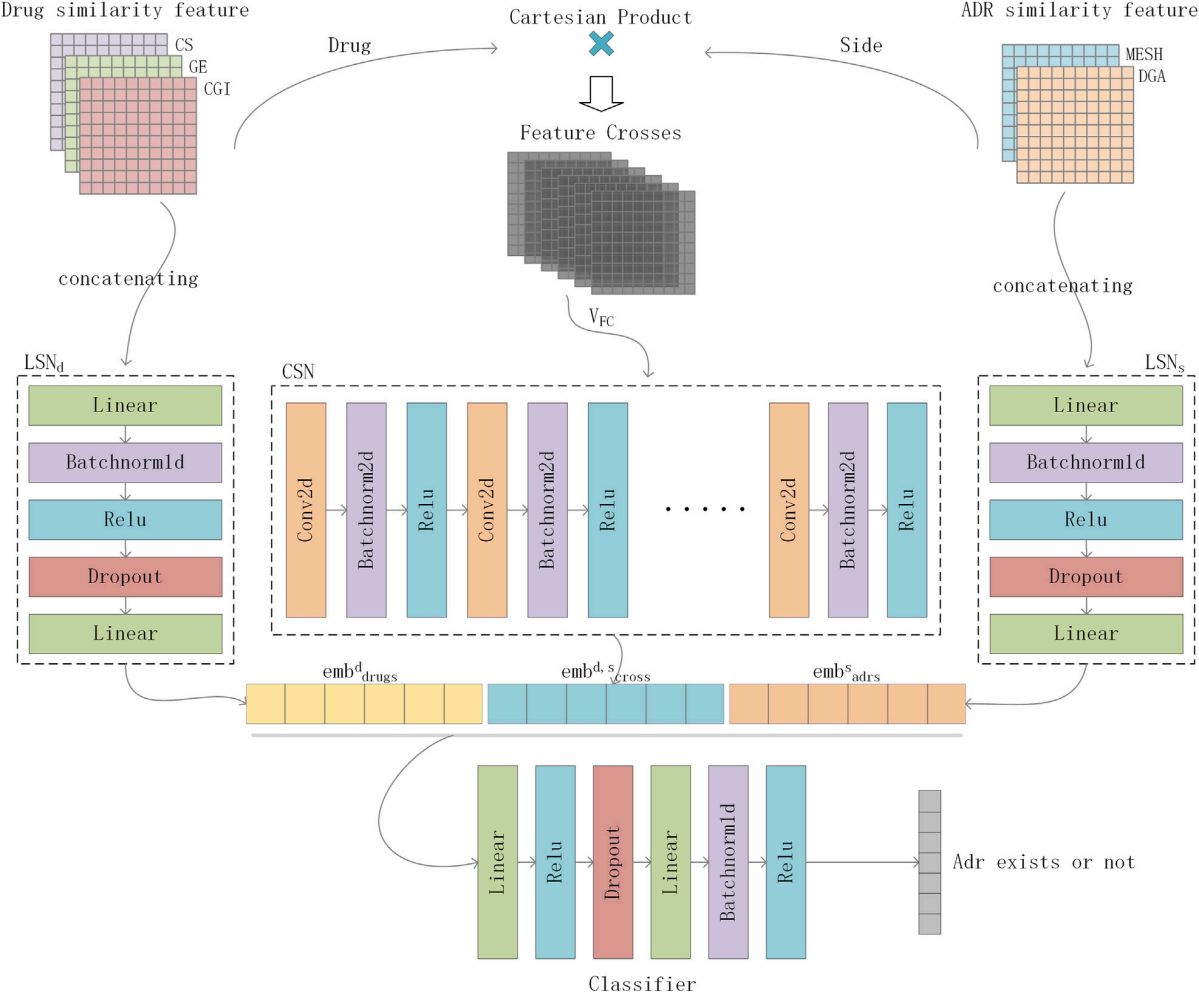
Flowchart of DGANet in ADR prediction. For a drug-side effect pair, DGANet employs two Linear Subnetworks ( ${LSN}_{d}$ for drug d and ${LSN}_{s}$ for side effect s ) and one Convolutional Neural Network with a Subnetwork (CSN) for feature crosses $V_{FCs}$ .

To effectively integrate similarity information from multiple data sources for ADR prediction, we proposed DGANet, a CNN-based multi-label classification architecture that considers each label as an independent binary problem. Figure 3 depicts the architecture of DGANet.

For a drug-ADR pair, DGANet employs two Linear Subnetworks ( ${LSN}_{d}$ for drug d and ${LSN}_{s}$ for ADR s ) and one Convolutional Neural Network with a Subnetwork (CSN) for feature crosses $V_{FCs}$ . The two LSNs, ${LSN}_{d}$ and ${LSN}_{s}$ , share the same architecture. The LSN formula can be summarized as follows:

$$x_{m,k}=Linear\left({Dropout}^{\left(p\right)}\left(RelU\left({FC}^{n1}\left(CAT\left(x_{m},c_{k}\right)\right)\right)\right)\right)$$

where $x_{m,k}$ is the latent representation of m drugs or m ADRs with k different similarity features, which is the output of the LSN, ${FC}^{\left(n\right)}$ is a fully connected layer with n neurons, and ${Dropout}^{\left(p\right)}$ is a dropout layer with probability p . On the other hand, Linear and RelU represent linear and rectified linear unit activation functions, respectively, and CAT concatenates given feature vectors. We then obtained the vector embeddings of drugs, ${emb}_{drugs}$ , and ADRs, ${emb}_{adrs}$ . A six-layered CNN was used. Previously constructed feature crosses $V_{FCs}$ were fed into the CSN to learn the representation of feature crosses, ${emb}_{cross}$ . Finally, the vector embeddings, ${emb}_{drugs}$ , ${emb}_{adrs}$ , and ${emb}_{cross}$ constructed with ${LSN}_{d}$ , ${LSN}_{s},$ and CSN , were concatenated and fed into a multi-label classifier, using two fully connected layers, activation functions, and a dropout. Finally, our model generated a vector, and values >0 indicated a correlation between the drug and ADR. The output vector can be represented as follows:

$$y_{d,s}=Relu\left({FC}^{n2}\left({Dropout}^{\left(p\right)}\left(Relu\left({FC}^{n1}\left(CAT\left({emb}_{drugs}^{d},{emb}_{cross}^{d,s},{emb}_{adrs}^{s}\right)\right)\right)\right)\right)\right)$$

where $y_{d,s}$ is the output association of drug d and ADR s, ${FC}^{\left(n\right)}$ is a fully connected layer with n neurons, and ${Dropout}^{\left(p\right)}$ is a dropout layer with probability p , and ${emb}_{drugs}^{d}$ , ${emb}_{cross}^{d,s}$ , and ${emb}_{adrs}^{s}$ are the embedding vectors of ${LSN}_{d}$ , ${emb}_{cross}^{d,s}$ CSN , and ${LSN}_{s}$ , respectively.

For model optimization, we adopted the ZLPR function (Su et al., 2022) to calculate the errors between the predicted and true values. The concept considers the correlation between labels, yielding more comprehensive outcomes than binary relevance methods. The loss function formula was as follows:

$$L_{tlpr}=\log\left(e^{s_{0}}+\sum_{i\in \Omega_{neg}}e^{s_{i}}\right)+\log\left(e^{{-s}_{0}}+\sum_{j\in \Omega_{pos}}e^{-s_{j}}\right)$$

where $\Omega_{pos}$ is the set of positive labels, and $\Omega_{neg}$ is the set of negative labels, $s_{i}$ is the model output score of the *i*th category. And $s_{0}$ was set to 0 in our experiment.

Finally, the loss function $L_{tlpr}$ was optimized using the Adam algorithm, and the learning rate was set to 0.005.

## 3 Results

### 3.1 Statistical analysis

**FIGURE 4:**
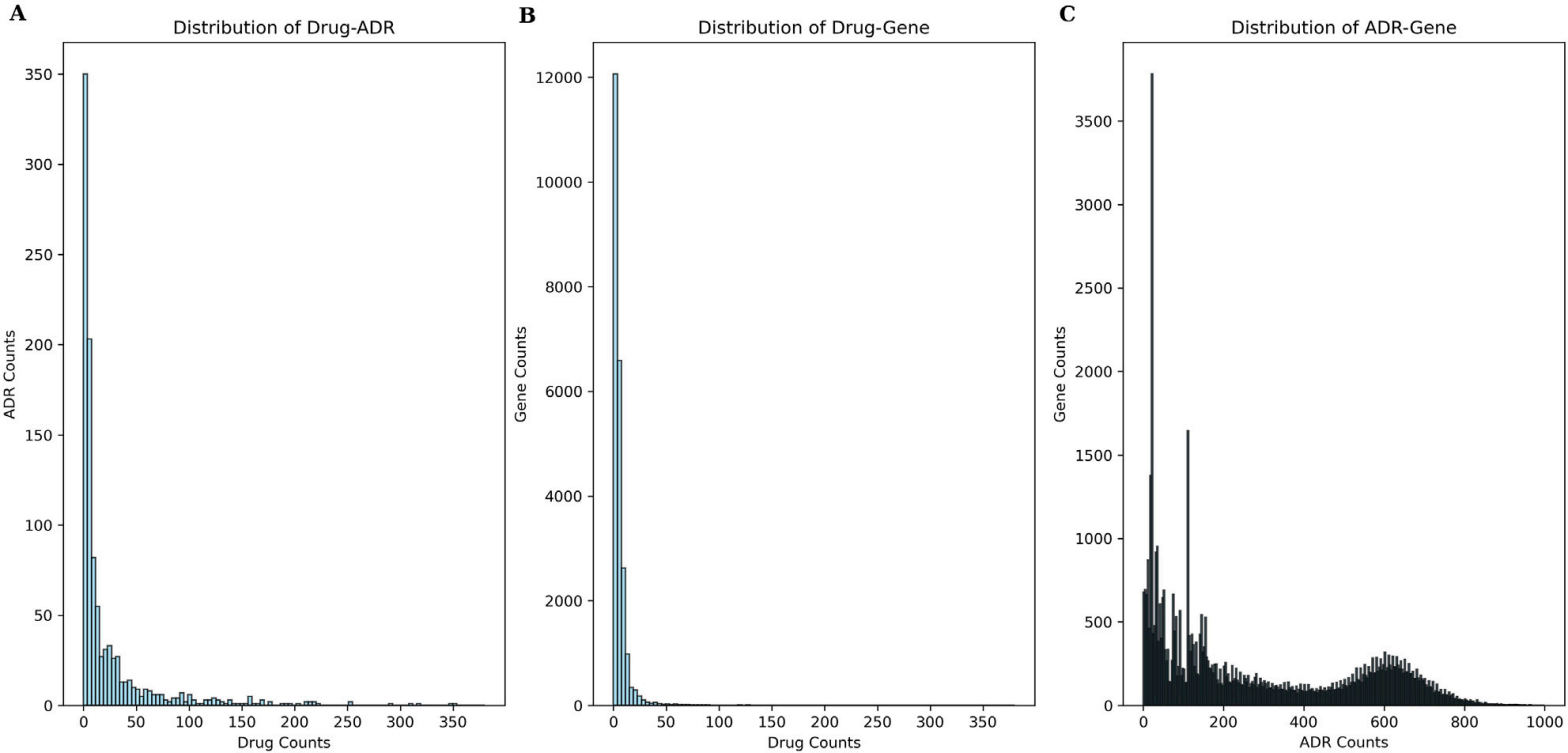
Frequency-rank distribution of experimental data. **(A)** presents the frequency-rank distribution of drug and related ADRs; **(B)** presents the frequency-rank distribution of drug and related genes; **(C)** presents the frequency-rank distribution of ADR and related genes.

As mentioned in Section 2.1, our benchmark dataset comprised 453 drugs, 1,091 ADRs, 101,257 CGIs with 23,644 genes, 15,087,041 GDAs with 53,968 genes, and 23,395 known drug-ADR pairs. The Drug-ADR, Drug-Gene, and ADR-Gene statistics exhibited a long-tail distribution (Figure 4). Specifically, few drugs accounted for a large proportion of all drug-ADR pairs and drug-gene interactions, whereas a large number of drugs were associated with only a small proportion of drug-ADR pairs and drug-gene interactions. Additionally, among these three statistics, the number of positive samples was far less than that of negative samples. A class-balanced sampling method was adopted to address the imbalance in the Drug-ADR dataset. The Jaccard Index was used to calculate the similarity between the Drug-Gene and ADR-Gene datasets.

### 3.2 Cross-validation test of ADR prediction for different combination features

The ADR prediction model was evaluated through 5-fold cross-validation. The Area Under the Receiver Operating Characteristic Curve (AUROC), Area Under the Precision-Recall Curve (AUPRC), Average Accuracy Percentage (Acc) and Matthews correlation coefficient (MCC) (Chicco and Jurman, 2023) metrics were used to assess our model’s performance in assigning the correct ADRs to individual drugs. Higher values in both metrics indicated better performance. In each fold, the model was trained using a randomly selected subset of 80% known drug-ADR associations and a matching number of randomly sampled non-associating pairs, with the remaining 20% utilized for testing. The AUROC and AUPRC values of the five folds were then averaged and used as a benchmark for comparison to the other algorithms and optimizing hyperparameters. Herein, seven different data settings for drugs [(i) CS + GE + CGI, (ii) CS + CGI, (iii) CS + GE, (iv) GE + CGI, (v) CS, (vi) CGI, and (vii) GE] and three different data settings for ADRs [(a) MESH + GDA, (b) MESH, and (c) GDA] were used to accomplish a fair comparison.

#### 3.2.1 Comparative analysis of genomic descriptors of drugs (GE and CGI)

**FIGURE 5:**
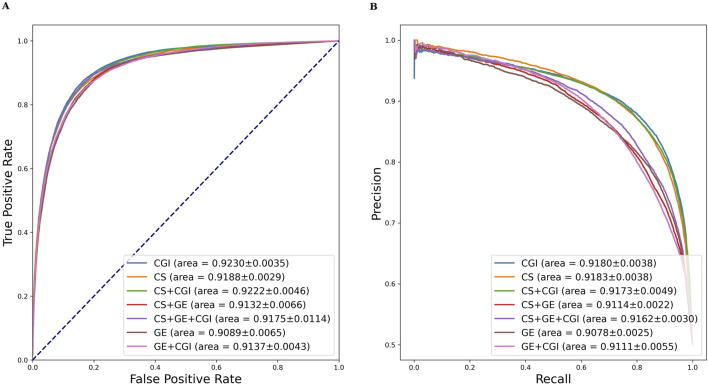
AUROC and AUPRC of DGANet with the fixed ADR feature setting (a) and seven different drug feature settings. **(A)** presents the AUROC curves of the comparison; **(B)** presents the AUPRC curves of the comparison.

A comparable result of GE and CGI was attained when the input feature of ADRs was set to MeSH + GDA (see Table 2). Figure 5 shows the AUROC and AUPRC values. According to the results, setting (vi; CGI) had the best performance (AUROC = 92.30 ± 0.35%, AUPRC = 91.80 ± 0.38%, MCC = 71.22 ± 0.66%, Acc = 85.57 ± 0.31), even better than that of setting (i) (CS + GE + CGI; AUROC = 91.75 ± 1.14%, AUPRC = 91.62 ± 0.30%, MCC = 70.49 ± 1.04%, Acc = 85.21 ± 0.54). This finding contradicts the widely held belief that adding more features to a model improves training accuracy. Compared to single feature settings [(v) CS and (vii) GE], the AUROC values of setting (vi; CGI) were higher by 0.42% and 1.41%, respectively. Furthermore, setting (ii; CS + CGI) performed better than setting (v; CS) and setting (iv; GE + CGI) performed better than setting (vii; GE). After adding CGI to CS and GE, the AUROC values increased by 0.34% and 0.48% respectively. Moreover, the AUROC and AUPRC values of setting (i; CS + GE + CGI) were 91.75% and 91.62%, respectively. These values were 0.43% and 0.48% higher than those of setting (iii; CS + GE). On the other hand, the AUROC scores of settings (iii), (iv), and (i) were lower than those of settings (v), (vi), and (ii) (by 0.56%, 0.93%, and 0.47%) respectively). These findings indicate that adding CGI improved model performance significantly in various situations, while adding GE decreased the AUROC and AUPRC values. In this regard, CGI is more informative than GE for ADR prediction, potentially because it contains interactions curated from various resources, providing more comprehensive drug information than GE, which is based solely on the experimental results of the LINC L1000 project.

**TABLE 2:** Evaluation results of different drug feature settings with ADR setting (a).

Settings	Drug feature	ADR feature	AUROC(%)	AUPRC (%)	Acc(%)	MCC(%)
i	CS + GE + CGI	MESH + GDA	91.75 ± 1.14	91.62 ± 0.30	85.21 ± 0.54	70.49 ± 1.04
ii	CS + CGI	MESH + GDA	92.22 ± 0.46	91.73 ± 0.49	85.01 ± 0.60	70.10 ± 1.20
iii	CS + GE	MESH + GDA	91.32 ± 0.66	91.14 ± 0.22	84.24 ± 0.41	68.58 ± 0.84
iv	GE + CGI	MESH + GDA	91.37 ± 0.43	91.11 ± 0.55	84.10 ± 0.37	68.26 ± 0.73
v	CS	MESH + GDA	91.88 ± 0.29	**91.83 ± 0.38**	84.82 ± 0.36	69.67 ± 0.72
vi	CGI	MESH + GDA	**92.30 ± 0.35**	91.80 ± 0.38	**85.57 ± 0.31**	**71.22 ± 0.66**
vii	GE	MESH + GDA	90.89 ± 0.65	90.78 ± 0.25	83.85 ± 0.46	67.88 ± 0.86

The best performance is highlighted in bold.

#### 3.2.2 Evaluation of the effect of GDA-based ADR similarity on DGANet

**FIGURE 6:**
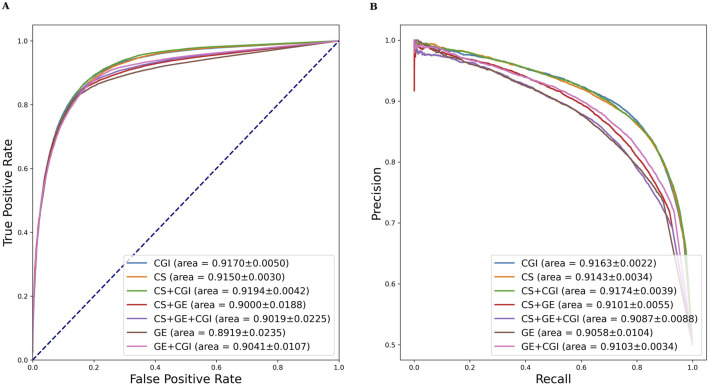
AUROC and AUPRC of DGANet with the fixed ADR feature setting (b) and seven different drug feature settings. **(A)** presents the AUROC curves of the comparison; **(B)** presents the AUPRC curves of the comparison.

**FIGURE 7:**
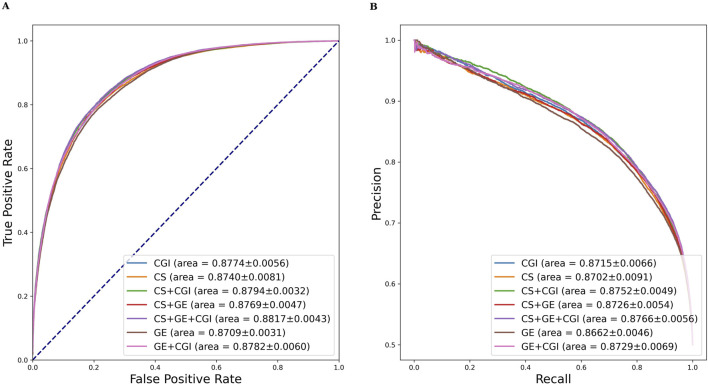
AUROC and AUPRC of DGANet with the fixed ADR feature setting (c) and seven different drug feature settings. **(A)** presents the AUROC curves of the comparison; **(B)** presents the AUPRC curves of the comparison.

Similar evaluation experiments were performed with ADR settings (b) and (c). The results are shown in Tables 3, 4. Figures 6, 7 show the AUROC and AUPRC, respectively. A comparison of Tables 2–4 revealed that ADR setting (a) had the highest scores, followed by setting (b), and setting (c) scored lowest. The gap was largest when the drug characteristics were set as CS + CGI. The AUROC values of setting (c) were 4.28% and 4.0% lower than those of settings (a) and (b), respectively. The AUROC and AUPRC values of Tables 2, 3 were both higher than 89.19%, whereas the highest AUROC and AUPRC scores in Table 4 were 88.17% and 86.62%, respectively. In addition, the MCC values in Tables 2, 3 exceeded 67.88%, whereas the MCC values in Table 4 were lower than 60%. Furthermore, MeSH was superior to GDA, with the integration of MeSH and GDA achieving the best outcome. In conclusion, GDA could improve the ADR prediction performance but had less information than the semantic similarity of ARs themselves.

**TABLE 3:** Evaluation results of different drug feature settings with ADR setting (b).

Settings	Drug feature	ADR feature	AUROC(%)	AUPRC(%)	Acc(%)	MCC(%)
i	CS + GE + CGI	MESH	90.19 ± 2.25	90.87 ± 0.88	84.50 ± 0.80	69.08 ± 1.63
ii	CS + CGI	MESH	**91.94 ± 0.42**	**91.74 ± 0.39**	84.90 ± 0.40	69.86 ± 0.82
iii	CS + GE	MESH	90.00 ± 1.88	91.01 ± 0.55	84.39 ± 0.46	68.83 ± 0.92
iv	GE + CGI	MESH	90.41 ± 1.07	91.03 ± 0.34	84.15 ± 0.25	68.38 ± 0.49
v	CS	MESH	91.50 ± 0.30	91.43 ± 0.34	84.48 ± 0.35	69.06 ± 0.72
vi	CGI	MESH	91.70 ± 0.50	91.63 ± 0.22	**84.93 ± 0.29**	**69.93 ± 0.58**
vii	GE	MESH	89.19 ± 2.35	90.58 ± 1.04	84.11 ± 0.79	68.29 ± 1.55

The best performance is highlighted in bold.

**TABLE 4:** Evaluation results of different drug feature settings with ADR setting (c).

Settings	Drug feature	ADR feature	AUROC(%)	AUPRC(%)	Acc(%)	MCC(%)
i	CS + GE + CGI	GDA	**88.17 ± 0.43**	**87.66 ± 0.56**	**79.90 ± 0.43**	**59.98 ± 0.92**
ii	CS + CGI	GDA	87.94 ± 0.32	87.52 ± 0.49	79.80 ± 0.34	59.83 ± 0.73
iii	CS + GE	GDA	87.69 ± 0.47	87.26 ± 0.54	79.34 ± 0.45	58.74 ± 0.88
iv	GE + CGI	GDA	87.82 ± 0.60	87.29 ± 0.69	79.40 ± 0.72	58.94 ± 1.38
v	CS	GDA	87.40 ± 0.81	87.02 ± 0.91	79.32 ± 0.98	58.68 ± 1.95
vi	CGI	GDA	87.74 ± 0.56	87.15 ± 0.66	79.46 ± 0.68	59.09 ± 1.30
vii	GE	GDA	87.09 ± 0.31	86.62 ± 0.46	78.77 ± 0.25	57.57 ± 0.50

The best performance is highlighted in bold.

### 3.3 Performance improvement in ADR prediction by adding similarity based on existing DSAs

Multiple studies (Poleksic and Xie, 2018; Zhao et al., 2022; Zhao et al., 2021) have recently shown that embedding neighborhood similarity of known DSAs can improve ADR prediction accuracy. However, this approach could easily lead to train-test contamination. Herein, the samples in the test set were set to zero before feature construction in each fold to avoid train-test contamination. In this regard, the model could learn nothing about the test set in the training phase. The results are shown in Tables 5. Compared to the results in Table 2, the top 3 settings (ii), (v), and (vi) exhibited increased AUROC (by 0.77%, 0.54%, and 0.44%, respectively), AUPRC (by 0.85%, 0.75%, and 0.85%, respectively), Acc (by 0.77%, 0.54%, and 0.44%, respectively) and MCC (by 0.77%, 0.54%, and 0.44%, respectively) values after adding similarity based on existing DSAs. The AUROC and AUPRC are presented in the Supplementary Figures S1–S3 illustrates the learning rate curves of our model. This finding indicates that integrating neighborhood similarity associated with known DSAs can improve the model’s performance. Notably, this method relies significantly on existing drug-ADR associations, and the information on new developed drugs and ADRs may be incomplete. Nonetheless, it still demonstrates high practicality and accuracy in predicting drug reuse ADRs and severe rare ADRs (Poleksic and Xie, 2018).

**TABLE 5:** Evaluation results of DGANet after adding existing drug-ADR associations to predict new ADRs.

Settings	Drug feature	ADR feature	AUROC(%)	AUPRC (%)	Acc(%)	MCC(%)
i	CS + GE + CGI + DSAs	MESH + GDA + DSAs	92.66 ± 0.50	92.39 ± 1.03	**85.95 ± 0.56**	**71.95 ± 1.16**
ii	CS + CGI + DSAs	MESH + GDA + DSAs	**92.76 ± 0.37**	92.49 ± 0.52	85.89 ± 0.33	71.84 ± 0.64
iii	CS + GE + DSAs	MESH + GDA + DSAs	92.49 ± 0.64	92.37 ± 0.72	85.43 ± 0.84	70.92 ± 1.69
iv	GE + CGI + DSAs	MESH + GDA + DSAs	92.55 ± 0.28	92.26 ± 0.49	85.65 ± 0.28	71.38 ± 0.57
v	CS + DSAs	MESH + GDA + DSAs	92.68 ± 0.31	92.58 ± 0.45	85.77 ± 0.49	71.64 ± 0.99
vi	CGI + DSAs	MESH + GDA + DSAs	92.74 ± 0.38	**92.65 ± 0.50**	85.78 ± 0.47	71.60 ± 0.94
vii	GE + DSAs	MESH + GDA + DSAs	92.26 ± 1.42	92.30 ± 1.03	85.63 ± 1.06	71.31 ± 2.13

The best performance is highlighted in bold.

### 3.4 Performance comparison between DGANet and the state-of-the-art ADR prediction models with pharmacogenoimic features

To evaluate the performance of the DGANet model, we compared it with three state-of-the-art methods with pharmacogenomic features, including Wang’s method (Wang et al., 2016), MMNN. Sum (Üner et al., 2023), DruGNN (Bongini et al., 2023) and BiMPADR (Li et al., 2024). Table 6 shows the comparison results. In Wang’s method and MMNN. Sum, ADRs associated with fewer than ten drugs were excluded, and the remaining drug-ADR datasets used in experiments remained imbalanced, leading to significantly lower AUPRC scores compared to AUROC scores. Our method (DGANet) addressed this imbalance by employing class-balanced sampling to achieve a balanced train and test dataset. Despite utilizing a smaller number of drugs and ADRs, our method still demonstrated notable improvements of 4.6% and 5.06% in AUROC score, respectively, with and without the incorporation of neighborhood similarity based on existing DSAs. While DGANet’s overall accuracy (85.95%) was slightly lower than DruGNN (86.3%), which was achieved among common ADRs (each drug may cause over 360 ADRs), DGANet’s accuracy surpassed the other Acc scores achieved by DruGNN when dealing with less common ADRs. Compared to BiMPADR, DGANet exhibited significant improvements of 3.36% and 4.05% in AUROC score and overall accuracy, respectively.

**TABLE 6:** Performance comparison of different models in the ADR prediction task with pharmacogenomics data.

Dataset	Model	Drug Features	ADR Features	#Drug /ADR	AUROC (%)	AUPRC (%)	ACC (%)
LINCS L1000 &SIDER	Wang et al.’s Method	GO + CS	—	791/1,053	85.40	—	—
	MMNN.Sum	CS+[GEX, META]	—	791/1,053	87.70	59.20	—
STITCH& SIDER	DruGNN	Drug-Gene graph	—	1,341/360	—	—	**86.30**
LINCS L1000 &SIDER& ADReCS	BiMPADR	Drug fingerprints +GE	ADR-Gene association	656/751	89.4	—	81.9
LINCS L1000 &SIDER&CTD	DGANet	CGI	MESH + GDA	453/1,019	92.30	91.87	85.57
		CS + GE +DSAs	MESH + GDA + DSAs	453/1,019	**92.76**	**92.49**	85.95

The best performance is highlighted in bold.

### 3.5 Literature evidence supports high ranked drug-induced ADRs

To further assess the performance of DGANet in identifying potential drug-ADR associations, case studies on the top 20 candidate drug-ADR associations unrecorded in SIDER were collected for validation and analysis (Supplementary Table S3). Moreover, we found that several indications were mistakenly predicted as ADR, such as Misoprostol and Pruritus. Among the 20 drug-ADR associations, 19 were included in MetaADEDB (Yu et al., 2021a) and OFFSIDES (Tatonetti et al., 2012), suggesting that the drug-induced ADRs were indeed associated with the corresponding drugs. And we collected the related genes from CTD database for 10 drug-ADR pairs, which might contribute to the occurrence of ADRs. The drug-induced ADRs labeled “Literature” were reported by published literature, indicating that they were not recorded in the adverse reaction databases, but their association has been reported previously (Yu et al., 2021b).

## 4 Discussion

Pharmacogenomics incorporates genomic profiling to identify biomarkers based on relevant genotype–phenotype interactions that can predict drug response and risk of ADRs. Using pharmacogenomics to predict adverse reactions can help improve the safety and effectiveness of medical care. To address the growing volume of complex, open-source pharmacogenomics data, artificial intelligence (AI) algorithms capable of large-scale computation and high-performance statistical analysis are essential. This paper introduces deep learning methods into the exploratory research of pharmacogenomics data analysis, leveraging the renowned CTD database, and proposes two characteristics for drugs and ADRs (CGI and GDA) and an intelligent prediction model (DGANet) for ADRs prediction based on the characteristics of pharmacogenomics data.

Initially, we curated a benchmark dataset comprising 453 drugs, 1,091 ADRs, 101,257 CGIs with 23,644 genes, 15,087,041 GDAs with 53,968 genes, and 23,395 known drug-ADR pairs from SIDER, LINCS L1000, CTD, PubChem, and MeSH source databases. Notably, CGIs and GDAs are the characteristic expression patterns we initially proposed to represent the relationships between drugs and genes, as well as adverse reactions and genes. In the evaluation experiments of ADR prediction, we compared these two pharmacogenomics features with traditional drug features (such as GE and CS). The five-fold cross-validation experimental results demonstrated that both of these pharmacogenomics features exhibit a significant tendency to enhance the predictive performance of ADRs. Particularly, CGI consistently outperformed the widely used drug features (GE and CS) in our experiments, and CGI could enhance model performance when combined with other drug features (Tables 2–5). Generally, the combination of CS and CGI achieved the highest AUROC and AUPRC values. However, we also observed some variations. In Table 2, the scores of CGI even surpassed those of the combined CS and CGI. We posit that this discrepancy might be attributed to the presence of coincidental information extracted from the same research between CGI and GDA.

Secondly, we proposed an intelligent model (DGANet) for predicting ADRs which achieved pharmacogenomics information fusion across all of drug, genomic and ADR features automatically. Compared with several state-of-the-art models based on different fusion methods of pharmacogenomics features and classifiers, the DGANet exhibited the highest performance in AUROC and AUPRC (Table 6). Case studies provide specific examples that further demonstrate the validity and practicality of the GDANet (Supplementary Table S3).

Despite being a preliminary study, our proposed DGANet model still has room for improvement in terms of accuracy and effectiveness. One limitation is DGANet’s inability to fully elucidate the diverse nature of drug-gene interactions and gene-disease associations, which is critical as the complex biological processes underlying drug responses are heavily influenced by specific gene expression variations. Another limitation is the lack of a comprehensive description of biological diversity within the currently utilized pharmacogenomics data, which poses a significant obstacle for all ADR prediction research methods.

## 5 Conclusion

In this study, we proposed DGANet, a new CNN-based model that integrates CGI and drug-perturbed GE changes (GE) into drug feature, GDA into ADR feature, and has achieved compelling results. The result showed that the two noval characteristics (CGI and GDA) we proposed both have an enhancing effect on the model. However, this study represents a preliminary investigation. By leveraging pharmacogenomics information and predicting adverse drug reactions, DGANet contributes to understanding how drugs influence gene expression and biological pathways that may lead to adverse reactions, offering valuable insights for drug safety research. Nevertheless, its mechanism is still unclear and further research is needed. More factors related to ADRs, such as gender, phenotype, dosage, etc., should be taken into account. With the latest advances in genomics and precision medicine, as well as regulatory guidance in pharmacogenomics, we believe that pharmacogenomics biomarkers will become increasingly common in all therapeutic fields. We anticipate that with ongoing data updates and the expansion of available databases, a richer pool of pharmacogenomics information will become accessible for future research into ADR prediction methods. In future studies, we plan to integrate more pharmacogenomics information into our model, such as Chemical–GO enriched associations, Chemical–pathway enriched associations and so on. The aim will be to find a more effective algorithm and corresponding feature construction methods to predict ADR more effectively and accurately.

## Data Availability

Publicly available datasets were analyzed in this study. This data can be found here: https://github.com/hemingxiu/DGANet.

## Abbreviations

ADR, Adverse Drug Reactions; DDI, Drug‒Drug Interaction; CS, Chemical Structure; GE, Gene Expression; GDA, Gene-Disease Association; CGI, Chemical-Gene Interactions; DSA, Drug-Side Effect Association; LSN, Linear Subnetworks; CSN, Convolutional Neural Network with a Subnetwork; AUROC, Area under the receiver operating characteristic curve; AUPRC, Area under the precision-recall curve.

## References

[B1] Amberger J. S. Hamosh A. (2017). Searching online mendelian inheritance in man (OMIM): a knowledgebase of human genes and genetic phenotypes. Curr. Protoc. Bioinforma. 58, 1. 10.1002/cpbi.27 PMC5662200 28654725

[B2] Bodenreider O. (2004). The unified Medical Language System (UMLS): integrating biomedical terminology. Nucleic Acids Res. 32, D267–D270. 10.1093/nar/gkh061 14681409 PMC308795

[B3] Bongini P. Scarselli F. Bianchini M. Dimitri G. M. Pancino N. Lió P. (2023). Modular multi–source prediction of drug side–effects with DruGNN. IEEE/ACM Trans. Comput. Biol. Bioinforma. 20, 1211–1220. 10.1109/TCBB.2022.3175362 35576419

[B4] Cacabelos R. Cacabelos N. Carril J. C. (2019). The role of pharmacogenomics in adverse drug reactions. Expert Rev. Clin. Pharmacol. 12, 407–442. 10.1080/17512433.2019.1597706 30916581

[B5] Chicco D. Jurman G. (2023). The Matthews correlation coefficient (MCC) should replace the ROC AUC as the standard metric for assessing binary classification. BioData Min. 16 (1), 4. 10.1186/s13040-023-00322-4 36800973 PMC9938573

[B6] Clark N. R. Hu K. S. Feldmann A. S. Kou Y. Chen E. Y. Duan Q. (2014). The characteristic direction: a geometrical approach to identify differentially expressed genes. BMC Bioinforma. 15, 79–16. 10.1186/1471-2105-15-79 PMC4000056 24650281

[B7] Das P. Mazumder D. H. (2023). An extensive survey on the use of supervised machine learning techniques in the past two decades for prediction of drug side effects. Artif. Intell. Rev. 56, 9809–9836. 10.1007/s10462-023-10413-7 PMC9930028 36819660

[B8] Davis A. P. Wiegers T. C. Johnson R. J. Sciaky D. Wiegers J. Mattingly C. J. (2023). Comparative Toxicogenomics database (CTD): update 2023. Nucleic Acids Res. 51, D1257–D1262. 10.1093/nar/gkac833 36169237 PMC9825590

[B9] Durant J. L. Leland B. A. Henry D. R. Nourse J. G. (2002). Reoptimization of MDL keys for use in drug discovery. Am. Chem. Soc. 42, 1273–1280. 10.1021/ci010132r 12444722

[B10] Fernandez-Llimos F. Minguet F. Salgado T. M. (2017). New pharmacy-specific medical subject Headings included in the 2017 database. Am. J. Health-System Pharm. 74, 1128–1129. 10.2146/ajhp170046 28743776

[B11] Geurts P. Ernst D. Wehenkel L. (2006). Extremely randomized trees. Mach. Learn. 63 (1), 3–42. 10.1007/s10994-006-6226-1

[B12] Huang L.-H. He Q. S. Liu K. Cheng J. Zhong M. D. Chen L. S. (2018). ADReCS-Target: target profiles for aiding drug safety research and application. Nucleic Acids Res. 46 (Database issue), D911-D917–D917. 10.1093/nar/gkx899 30053268 PMC5753178

[B13] Kim S. Chen J. Cheng T. Gindulyte A. He J. He S. (2019). PubChem 2019 update: improved access to chemical data. Nucleic Acids Res. 47, D1102-D1109–D1109. 10.1093/nar/gky1033 30371825 PMC6324075

[B14] Köhler S. Vasilevsky N. A. Engelstad M. Foster E. McMurry J. Aymé S. (2017). The human phenotype Ontology in 2017. Nucleic Acids Res. 45, D865-D876–D876. 10.1093/nar/gkw1039 27899602 PMC5210535

[B15] Kuhn M. Letunic I. Jensen L. J. Bork P. (2016). The SIDER database of drugs and side effects. Nucleic Acids Res. 44, D1075–D1079. 10.1093/nar/gkv1075 26481350 PMC4702794

[B16] Kuhn M. von Mering C. Campillos M. Jensen L. J. Bork P. (2008). STITCH: interaction networks of chemicals and proteins. Nucleic Acids Res. 36, D684–D688. 10.1093/nar/gkm795 18084021 PMC2238848

[B17] Landrum G. (2024). rdkit/rdkit: 2024_09_1 (Q3 2024) Release. Zenodo. 10.5281/zenodo.13848108

[B18] Li S. Zhang L. Wang L. Ji J. He J. Zheng X. (2024). BiMPADR: a deep learning framework for predicting adverse drug reactions in new drugs. Mol. Basel, Switz. 29 (8), 1784. 10.3390/molecules29081784 PMC11051887 38675604

[B19] Lian J. Zhou X. Zhang F. Chen Z. Xie X. Sun G. (2018). “xDeepFM: combining explicit and implicit feature interactions for recommender systems,” in Proceedings of the 24th ACM SIGKDD international conference on knowledge discovery and data mining (New York, NY, USA: Association for Computing Machinery), 1754–1763. 10.1145/3219819.3220023

[B20] Micaglio E. Locati E. T. Monasky M. M. Romani F. Heilbron F. Pappone C. (2021). Role of pharmacogenetics in adverse drug reactions: an update towards personalized medicine. Front. Pharmacol. 12, 651720. 10.3389/fphar.2021.651720 33995067 PMC8120428

[B21] Pathan M. Londhe M. Jadhav D. (2018). “Drug-induced diseases: prevention, detection, and management,” in *Drug-induced diseases*, (American society of health-system pharmacists), 49–50. Available at: https://publications.ashp.org/display/book/9781585285310/9781585285310.xml (Accessed May 8, 2024).

[B22] Pirmohamed M. (2023). Pharmacogenomics: current status and future perspectives. Nat. Rev. Genet. 24, 350–362. 10.1038/s41576-022-00572-8 36707729

[B23] Poleksic A. Xie L. (2018). Predicting serious rare adverse reactions of novel chemicals. Bioinformatics 34, 2835–2842. 10.1093/bioinformatics/bty193 29617731 PMC6084596

[B24] Raja K. Patrick M. Elder J. T. Tsoi L. C. (2017). Machine learning workflow to enhance predictions of Adverse Drug Reactions (ADRs) through drug-gene interactions: application to drugs for cutaneous diseases. Sci. Rep. 7, 3690. 10.1038/s41598-017-03914-3 28623363 PMC5473874

[B25] Research C. for D. E. (2022). Finding and learning about side effects (adverse reactions). FDA. Available at: https://www.fda.gov/drugs/information-consumers-and-patients-drugs/finding-and-learning-about-side-effects-adverse-reactions.

[B26] Satapornpong P. Vorasatit L. John S. (2024). Advances in clinical pharmacogenomics and prevention of severe cutaneous adverse drug reactions in the era of precision medicine. IntechOpen. 10.5772/intechopen.1003691

[B27] Sedlmayr M. Zoch M. Wolfien M. Peng Y. Ahmadi N. (2024). OMOP CDM can facilitate data-driven studies for cancer prediction. Int. J. Mol. Sci. 23, 1–11. 10.3390/ijms231911834 PMC9569469 36233137

[B28] Shankar S. Bhandari I. Okou D. T. Srinivasa G. Athri P. (2021). Predicting adverse drug reactions of two-drug combinations using structural and transcriptomic drug representations to train an artificial neural network. Chem. Biol. and Drug Des. 97, 665–673. 10.1111/cbdd.13802 33006799

[B29] Su J. Zhu M. Murtadha A. Pan S. Wen B. Liu Y. (2022). ZLPR: a novel loss for multi-label classification. Available at: https://arxiv.longhoe.net/abs/2208.02955v1 (Accessed May 8, 2024).

[B30] Subramanian A. Narayan R. Corsello S. M. Peck D. D. Natoli T. E. Lu X. (2017). A next generation connectivity map: L1000 platform and the first 1,000,000 profiles. Cell 171, 1437–1452. 10.1016/j.cell.2017.10.049 29195078 PMC5990023

[B31] Tatonetti N. P. Ye P. P. Daneshjou R. Altman R. B. (2012). Data-driven prediction of drug effects and interactions. Sci. Transl. Med. 4, 125ra31. 10.1126/scitranslmed.3003377 PMC3382018 22422992

[B32] Üner O. C. Kuru H. I. Cinbis R. G. Tastan O. Cicek A. E. (2023). DeepSide: a deep learning approach for drug side effect prediction. IEEE/ACM Trans. Comput. Biol. Bioinforma. 20, 330–339. 10.1109/TCBB.2022.3141103 34995191

[B33] Wang Z. Clark N. R. Ma’ayan A. (2016). Drug-induced adverse events prediction with the LINCS L1000 data. Bioinformatics 32, 2338–2345. 10.1093/bioinformatics/btw168 27153606 PMC4965635

[B34] Yu R. J. Krantz M. S. Phillips E. J. Stone C. A. (2021a). Emerging causes of drug-induced anaphylaxis: a review of anaphylaxis-associated reports in the fda adverse event reporting system (faers). J. Allergy Clin. Immunol. Pract. 9, 819–829.e2. 10.1016/j.jaip.2020.09.021 32992044 PMC7870524

[B35] Yu Z. Wu Z. Li W. Liu G. Tang Y. (2021b). MetaADEDB 2.0: a comprehensive database on adverse drug events. Bioinformatics 37, 2221–2222. 10.1093/bioinformatics/btaa973 33306787

[B36] Zhang J. D. Sach-Peltason L. Kramer C. Wang K. Ebeling M. (2020). Multiscale modelling of drug mechanism and safety. Drug Discov. Today 25 (3), 519–534. 10.1016/j.drudis.2019.12.009 31899257

[B37] Zhao H. Wang S. Zheng K. Zhao Q. Zhu F. Wang J. (2022). A similarity-based deep learning approach for determining the frequencies of drug side effects. Briefings Bioinforma. 23, bbab449. 10.1093/bib/bbab449 34718402

[B38] Zhao H. Zheng K. Li Y. Wang J. (2021). A novel graph attention model for predicting frequencies of drug–side effects from multi-view data. Briefings Bioinforma. 22, bbab239. 10.1093/bib/bbab239 34213525

[B39] Zhou Z.-W. Chen X. W. Sneed K. B. Yang Y. X. Zhang X. He Z. X. (2015). Clinical association between pharmacogenomics and adverse drug reactions. Drugs 75 (6), 589–631. 10.1007/s40265-015-0375-0 25895462

