# Supplementary material for: Prediction of adverse drug reactions based on pharmacogenomics combination features: a preliminary study
Source: Front Pharmacol. 2025 Mar 10;16:1448106. doi: 10.3389/fphar.2025.1448106 (PMC11931068; doi:10.3389/fphar.2025.1448106)
Supplement: Supplementary file 1 [file DataSheet1.pdf]

Supplementary Material

1 Supplementary Figures and Tables

1.1 Supplementary Figures

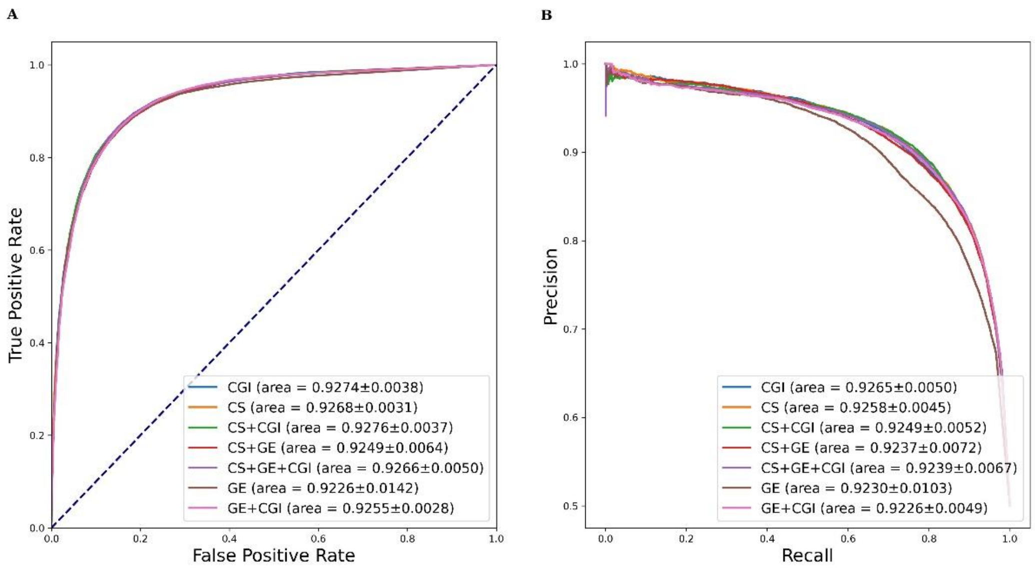

Supplementary Figure 1. The AUROC and AUPRC of DGANet with known DSAs, the fixed ADR feature setting (a) and seven different drug feature settings. Panel (A) presents the AUROC curves of the comparison; Panel (B) presents the AUPRC curves of the comparison.

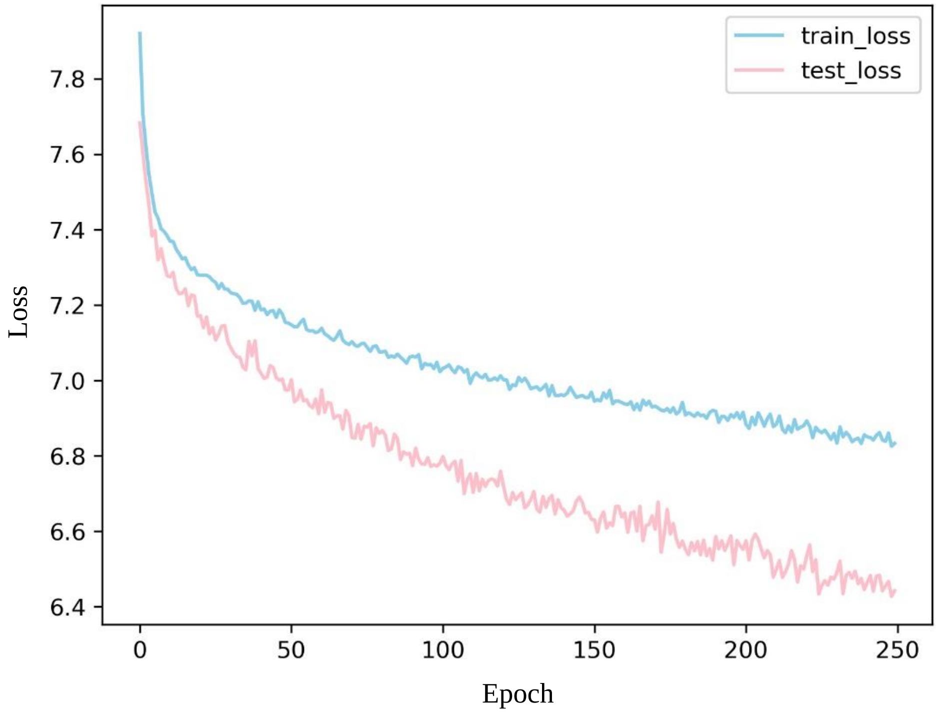

Supplementary Figure 2. The learning rate curves of DGANet (with known DSAs, drug feature is set to (ii) and ADR feature is set to (a)). The learning curve tends to stabilize at the 250th epoch.

1.2 Supplementary Tables

Supplementary Table S3. Top 20 ranked drug-induced ADRs predicted by DGANet.

No.	Drug	ADR	Related Genes	Predict score	Literature evidence
**1**	Pravastatin	Urticaria	*IL1B*, *TNF*	9.572176	MetaADEDB, OFFSIDES
**2**	Meloxicam	Urticaria	*IL1B*, *TNF*	9.571867	MetaADEDB, OFFSIDES
**3**	Levetiracetam	Angioedema		9.221045	MetaADEDB, OFFSIDES
**4**	Perindopril	Nausea	*TNF*	9.176565	MetaADEDB, OFFSIDES
**5**	Simvastatin	Hypersensitivity	*CASP1*, *CCL2*, *CCL20*, *CCL3*, *CCL4*, *CCR1*, *CCR2*, *CD40LG*, *CXCL1*, *CXCL15*, *CXCL5*, *CXCR2*, *CYP1A1*, *FOXP3*, *HLA-DQB1*, *IFNG*, *IL13*, *IL16*, *IL17A*, *IL18*,	9.123637	MetaADEDB, OFFSIDES

			IL1B, IL1R2, IL4, IL6, ITGB2, MTHFR, SELP, SPP1, TGFB1, TNF, TNFRSF1A, TNFRSF1B		
**6**	Acamprosate	Myalgia		9.113294	MetaADEDB, OFFSIDES
**7**	Carvedilol tartrate	Exanthema	*IL1RN*	9.105224	MetaADEDB, OFFSIDES
**8**	Enalaprilat	Angioedema		9.098155	MetaADEDB, OFFSIDES
**9**	Misoprostol	Pruritus	*TAC1*	9.054532	MetaADEDB, OFFSIDES
**10**	Memantine	Myalgia		9.027507	MetaADEDB, OFFSIDES
**11**	Atorvastatin	Urticaria	*ICAM1, IL18, IL1B, MPO, SELE, TGFB1, TNF, VCAM1*	8.987735	MetaADEDB, OFFSIDES
**12**	Fenofibrate	Hypersensitivity	*ALB,CCL2,CD40LG,CX3CL1,CYP1A1,IFNG,IL1B,IL6,MYLK,TGFB1,TNF*	8.969686	MetaADEDB, OFFSIDES
**13**	Memantine	Asthenia		8.944061	MetaADEDB, OFFSIDES
**14**	Lisinopril	Anaphylaxis		8.927623	Literature (R. J. Yu et al., 2021)
**15**	Carvedilol tartrate	Pruritus	*ABCB11*	8.914467	MetaADEDB, OFFSIDES
**16**	Misoprostol	Abdominal Pain		8.9103565	MetaADEDB, OFFSIDES
**17**	Everolimus	Urticaria	*TNF*	8.879842	MetaADEDB, OFFSIDES
**18**	Fludarabine	Dyspepsia		8.857897	MetaADEDB, OFFSIDES
**19**	Carvedilol tartrate	Constipation		8.812408	MetaADEDB, OFFSIDES
**20**	Docetaxel	Pruritus		8.80261	MetaADEDB, OFFSIDES

References

Yu, R. J., Krantz, M. S., Phillips, E. J., and Stone, C. A. (2021a). Emerging Causes of Drug-Induced Anaphylaxis: A Review of Anaphylaxis-Associated Reports in the FDA Adverse Event Reporting System (FAERS). *J Allergy Clin Immunol Pract* 9, 819-829.e2. doi: 10.1016/j.jaip.2020.09.021
